# Effects of UV radiation on natural and synthetic materials

**DOI:** 10.1007/s43630-023-00377-6

**Published:** 2023-04-11

**Authors:** A. L. Andrady, A. M. Heikkilä, K. K. Pandey, L. S. Bruckman, C. C. White, M. Zhu, L. Zhu

**Affiliations:** 1 Department of Chemical and Biomolecular Engineering, North Carolina State University, Raleigh, NC USA; 2 Finnish Meteorological Institute, Helsinki, Finland; 3 Indian Academy of Wood Science, Bangalore, India; 4 Department of Materials Science and Engineering, Case Western Reserve University, Cleveland, OH USA; 5 Exponent Inc, Bowie, MD USA; 6 College of Materials Science and Engineering, Donghua University, Shanghai, China; 7 State Key Laboratory for Modification of Chemical Fibres and Polymer Materials, Donghua University, Shanghai, China

## Abstract

The deleterious effects of solar ultraviolet (UV) radiation on construction materials, especially wood and plastics, and the consequent impacts on their useful lifetimes, are well documented in scientific literature. Any future increase in solar UV radiation and ambient temperature due to climate change will therefore shorten service lifetimes of materials, which will require higher levels of stabilisation or other interventions to maintain their lifetimes at the present levels. The implementation of the Montreal Protocol and its amendments on substances that deplete the ozone layer, controls the solar UV-B radiation received on Earth. This current quadrennial assessment provides a comprehensive update on the deleterious effects of solar UV radiation on the durability of natural and synthetic materials, as well as recent innovations in better stabilising of materials against solar UV radiation-induced damage. Pertinent emerging technologies for wood and plastics used in construction, composite materials used in construction, textile fibres, comfort fabric, and photovoltaic materials, are addressed in detail. Also addressed are the trends in technology designed to increase sustainability via replacing toxic, unsustainable, legacy additives with ‘greener’ benign substitutes that may indirectly affect the UV stability of the redesigned materials. An emerging class of efficient photostabilisers are the nanoscale particles that include oxide fillers and nanocarbons used in high-performance composites, which provide good UV stability to materials. They also allow the design of UV-shielding fabric materials with impressive UV protection factors. An emerging environmental issue related to the photodegradation of plastics is the generation of ubiquitous micro-scale particles from plastic litter exposed to solar UV radiation.

**Graphical abstract:**

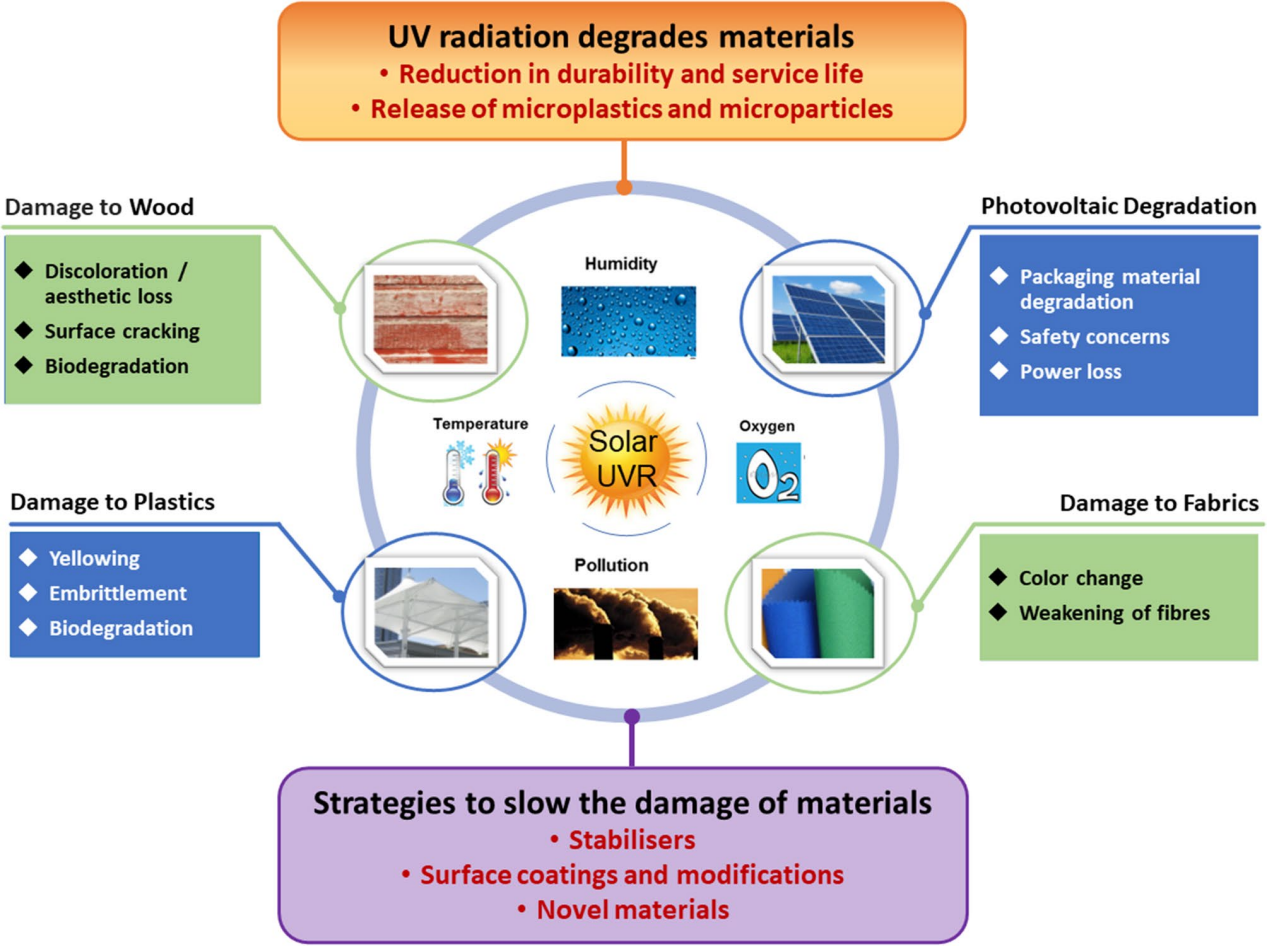

## Introduction

The Montreal Protocol and its Amendments over the last 35 years have successfully contributed to the stability of the stratospheric ozone layer, limiting the solar ultraviolet (UV) radiation, particularly the UV-B wavebands (280–315 nm) of terrestrial solar radiation reaching the Earth’s surface. Any future increase in solar UV radiation reaching the Earth, particularly the UV-B radiation, especially if accompanied by higher ambient temperatures, will shorten the service lifetimes of wood, plastics, and other organic materials. Given the low to moderate activation energies of photodegradation of these materials, even a small increase in the ambient temperature due to climate change, may accelerate degradation and shorten their service life. The presently available UV-stabilisation and coating technologies are expected to be adequate to mitigate these changes and maintain the service lifetimes of materials at the present level. However, elevated amounts of solar UV radiation in terrestrial solar radiation would require using correspondingly higher levels of UV stabilisers or more frequent surface treatment in the case of wood to ensure adequate service lifetimes. This strategy could invariably add significantly to the lifetime cost of materials. The materials industry is continuously researching more efficient and lower cost UV-stabiliser systems for wood and plastics. In recent years, this search has been increasingly guided by long-term sustainability considerations that encourage the preferred development of ‘greener’ additives, such as natural UV stabilisers in place of conventional synthetic additives for both plastics and wood [1]. Using nanoparticles (NPs) as stabiliser/fillers in coatings, plastics, or textile fibres, which results in efficient light-shielding at very low fractions, is an especially promising development in new stabilisers.

With the projected increase in world population, the demand for wood and plastic materials popularly used in building construction will increase in the medium term. Global production volumes for both these are the highest reported over the past 70 years. For instance, 2.03 billion m^3^ of industrial round wood processed in 2018 was mostly used as building materials [2]. Of the 359 million metric tons (MMTs) of plastic resins produced in 2018, about 30% was used in building applications [3]. With the number of buildings worldwide estimated to double by 2060 [4], with the addition of 230 billion m^2^ of new floor area (relative to 2017), the demand for construction materials will correspondingly increase. This increase will likely have significant environmental impacts at the global scale. The construction industry already accounts for 36% of energy expenditure and 37% of carbon dioxide (CO_2_) emissions globally [5]. Increased environmental sustainability in the industry will be critical in the coming decade. The trend towards sustainability is already apparent in the sector; the global investment in certified energy-efficient buildings increased by 11% in 2020, reflecting this trend.

Wood is a traditional, low-cost, and sustainable building material that has a relatively small carbon footprint relative to concretes, metals, or plastics [6]. For instance, a recent analysis illustrated the advantages of using wood over plastics as the material for fabricating pallets, a product with an annual global demand of 6.87 billion units [7]. Life cycle analysis (LCA) found wood to have a significant advantage over plastics in this application. Assuming incineration as the disposal method, the carbon footprint of a wood pallet was 0.34 kg of carbon dioxide equivalent (CO_2_-e) four times lower than that of a plastic unit [8]. A Finnish study [7] agreed with the finding, but found wood-plastic composites (WPC) to have even a lower carbon footprint relative to wood. Using recycled post-consumer plastics in the WPC further decreases emissions. A similar comparison between metal, poly(vinyl chloride) (PVC), and wood used in constructing window frames found the latter to have the least environmental costs as measured by embodied non-renewable energy and carbon emissions [9]. Findings from LCA studies can vary with location because of transportation and energy mix used. Therefore, they are not necessarily comparable across studies. These sustainability advantages make wood, especially the new ‘mass timber’ (or the engineered wood designed to compete with concrete or metal in strength), extremely attractive for use in buildings. Cross-laminated timber, a popular category of mass timber, was recently used to replace concrete in a high-rise building that is 18 stories high [10].

Materials used in building construction, transportation, and outdoor furniture are routinely exposed to solar UV radiation and undergo gradual photodegradation. The duration of their exposure to solar UV radiation determines the service life of these products such as composites used in vehicles, exterior panels in buildings, greenhouse glazing in agriculture, stadium seating, and synthetic turf. Photodegradation negatively affects appearance, compromises mechanical integrity, and encourages subsequent biodegradation, which all limit their service life [11]. With plastics, it is the UV-induced autocatalytic photo-oxidation reactions that result in surface discolouration or cracking accompanied by the loss of mechanical integrity over time. Photodamage to plastics, however, is mostly limited to a thin surface layer, resulting in damage modes such as yellowing, chalking, and cracking. As the base plastic resins are inherently photolabile, several groups of additives (UV stabilisers) are typically compounded with plastics intended for extended outdoor use, to mitigate the damaging effects of solar UV radiation [12–14]. These include UV absorbers, insoluble pigments that absorb or scatter UV radiation, and additives that inhibit oxidative reactions. Potent radical traps, such as tertiary amines of tertiary phenols, are often used to inhibit the free-radical photodegradation reactions [15, 16].

For instance, weathering of high-density polyethylene (HDPE) stabilised with UV absorbers, under accelerated weathering using a Xenon lamp (generally following ASTM G155), showed that the non-stabilised HDPE was embrittled, with its tensile strength extensibility reducing to 21%, in 300 h of exposure, while the UV-stabilised HDPE was stable up to 900 h of exposure [17]. However, the combination of a UV-absorber (0.15 wt%) with the radical-quencher hindered amine stabilisers (HALS) used at 0.45 wt%, yields synergistic stabilisation with little degradation up to 1300 h of exposure. Accelerated weathering of polypropylene (PP) with a HALS (0.5 wt%) and the UV screener, nano-ZnO (0.5 wt%), showed synergistic stabilisation [15]. However, the latter study used a high-intensity UV-A lamp. The effect of this high-intensity UV lamp with a narrow wavelength emission range (very different from spectral qualities of solar radiation) cannot be reasonably compared to that of natural weathering exposure. A recent investigation [18] on the natural weathering of UV-stabilised plastics over 4 years at a total UV irradiance of 1020 MJ m^−2^, ranked the durability of common plastic materials used in building. Their ranking was based on percentage retention of fracture strain after exposure. PP, poly(butylene terephthalate) (PBT), HDPE, and polycarbonate (PC) showed 47, 27, 20, and 17% retention of the property, respectively. The result, however, cannot be generalised as it will strongly depend on the additives, especially UV stabilisers, used in the plastics.

**Fig. 1:**
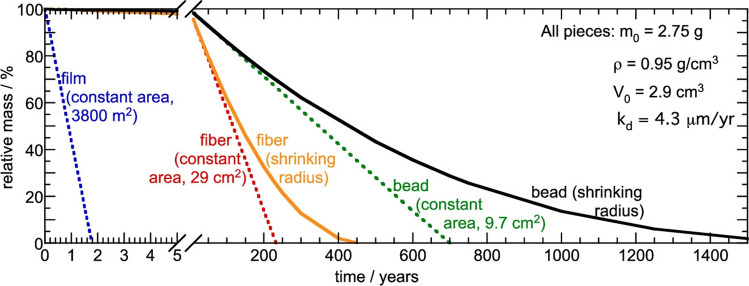
A comparison of predicted degradation profiles for high-density polyethylene (HDPE) pieces with the same mass (*m*_o_), density (*ρ*), and surface loss rate for different shapes (thin film, fibre, and bead). The dashed lines correspond to extrapolations assuming constant surface area; the solid lines correspond to a model that assumes a  shrinking radius, and therefore a surface area, decreasing with duration of degradative exposure. Figure from [19], CC BY 4.0

Studies on the photodegradation of plastics mostly focus on polyethylene (PE), polystyrene (PS), PVC, and poly(ethylene terephthalate) (PET). These plastics are found in solid waste stream, urban litter, fresh waters, and especially marine debris. A recent summary of the existing data on plastics degradation in air and aquatic environments in terms of mass or area loss rates was carried out [19, 20]. However, it relates primarily to fragmentation losses and perhaps included some photo-mineralisation. The latter process that converts the polymer into simple products such as CO_2_ and water, defines the environmentally desirable endpoint of biological degradation. Using a simple model, the study [19] demonstrates the geometry-dependence of the kinetics of photodegradation of HDPE (Fig. 1). Despite the simplifying assumptions used in the analysis, it highlights the significance of the geometric form of a plastic material in determining degradation rates, a factor often overlooked in other studies. Another model on the rate of surface area loss on degradation in environmental degradation is based on polymer characteristics, such as the hydrophobicity, glass transition temperature, and the fractional crystallinity [21].

The predominant type of plastic used in building construction is PVC, which is particularly susceptible to damage by solar UV radiation. The polymer undergoes photo-dehydrochlorination, releasing hydrogen chloride and yielding conjugated unsaturation. The PVC surface appears yellow to orange in colour on weathering, depending on the sequence lengths of the unsaturated units generated on the polymer molecules. Degradation of the PVC surface releases the opacifier, titanium dioxide (TiO_2_), compounded into the plastic and results in chalking or removal of the loose oxide on handling. However, it is the uneven discolouration of PVC products used in residential construction that determines their useful service life. Therefore, effective photostabilisers are critical to maintaining their outdoor service life under solar UV irradiation. In outdoor uses such as siding or panels, PVC is compounded with rutile titania as an opacifier and tin-based organic photostabilisers to control UV-induced degradation and thermal oxidation, respectively. Several studies investigated the use of tin-based stabilisers for this purpose. A novel tin-based stabiliser, tin-sulfadiazine complexes, at a 0.5 wt% in rigid PVC, reduced the dehydrochlorination rate constants to less than half that for control PVC samples in accelerated weathering studies [22]. Different organotin complexes with similar stabiliser function include fusidate [23], captopril [24], atenolol [25], mefenamic acid [26], naphthalene sulfonic acid [27], 4-methoxybenzoic acid [28], and carvedilol tin complexes [29]. While some of these show good stabiliser effectiveness, further development in terms of cost-effective manufacture and their environmental impacts will be needed before their commercialisation is considered.

In wood products, the photoreactions generally increase surface hydrophilicity that encourages subsequent biological degradation [30]. Using opaque or UV-absorbing surface coatings on wood ensures good stability under UV radiation [31]. However, the solvent-borne coatings contribute to the release of volatile organic chemicals (VOCs) into the atmosphere. Biodegradation, that also takes place concurrently on weathering, can be controlled by treating the wood thermally or chemically. Chemicals typically used in treated wood (chromated copper arsenate, creosotes, and chlorophenols), however, can leach into the environment. As with plastics, the emphasis on developing sustainable strategies also guides the criteria in selecting coatings and stabilisers for wood, with candidates for substitutes of legacy additives such as plasticisers, fillers, thermal stabilisers, colourants, and flame retardants. The incidental effects of these ‘greener’ substitute additives such as natural oil preservatives on the UV stability of wood still need to be evaluated.

### Building industry trends and materials use

Several emerging trends in the building industry will indirectly affect the mix of organic building materials to be used in future buildings. These may have important implications on their useful life under projected climate warming and increased UV radiation in areas of reduced cloud cover (Bernhard et al. [32]). There are two such trends apparent in the building industry that may indirectly impact the choice of materials and their durability. Among these are 3D printed homes and waste plastic utilisations in building technology. The trend in the building industry towards environmental sustainability encourages builders to qualify for Leadership in Energy and Environmental Design (LEED) [33] certification in the USA or Building Research Establishment Environmental Assessment Method (BREEAM) in Europe [34]. Carbon emissions in the sector are mostly due to the use of Portland cement and neither engineered wood products [35] nor plastic materials typically have comparably high carbon emissions. The ambitious LEED Zero program [33] aims at offsetting all energy, carbon emissions, and waste costs associated with future buildings.

A recent sustainable approach to building is 3D printing that promises lower cost, comparable durability, and increased design freedom, with at least the same durability as conventional-built homes [36]. At this time these structures are mostly based on inorganic materials (mortar, clay, concrete), but plastic-based 3D homes are feasible. At least two commercial operations, one using polyurethane–carbon fibre composites (PassivDom in Ukraine) and another using waste plastic as the primary material (Azure Homes, Los Angeles, CA) have started operations. With just $10 million revenue in 2020, the printed-home industry has a high compound annual growth rate of > 91% in the near term with revenues increasing to over $1 billion by 2028 [37]. Using large volumes of plastics in printed homes would require managing the large VOC emissions, associated occupational hygiene concerns, waste disposal issues, and water contamination. While the approach will conserve wood, the effect of solar UV radiation on the entire printed plastic structure will need to be carefully evaluated in their use.

The recycled plastic waste is formed into plastic blocks or bricks for building [38], especially in emerging economies [39]. The mixed plastic waste stream composed primarily of PE, PP, PS, and PET will continue to increase in step with the world population and provide a low-cost reliable raw material. Being mostly thermoplastic, plastic waste can be readily melted into low-cost geocomposites for drainage systems and infill material for geosynthetic-encased granular columns [40]. Not only do they provide excellent durability at a low cost, but they also attain very significant labour savings in building operations compared to conventional materials. However, the material must be rendered fire resistant and the possibility of outgassing from slow degradation reactions in bricks needs to be addressed, for the approach to be viable. Several commercial vendors offer such homes, including Conceptos Plásticos (Columbia) and Othalo (Norway). This trend will also reduce the demand for wood in buildings, contributing towards a more sustainable future.

### Greener additives used in plastics and wood

An integral aspect of the drive toward sustainable building materials is the replacement of toxic legacy additives that are harmful to the environment with ‘greener’ alternatives [41–43]. In selecting additives, including UV stabilisers [44] for plastics or coatings, increasing attention is being paid to minimising their potential ecotoxicity as these additives often leach out to contaminate the environment [45]. Wang et al. [46] reported the presence of UV absorbers at ~ 100 ng/g level in marine organisms including fish. In the plastics industry, this trend is illustrated by the phase-out of two classes of  additives, phthalate plasticiser, and brominated flame retardants, a process well underway in Europe, USA, and Japan. While greener additives selected for their functionality may improve the overall sustainability of plastics or coatings, their use may inadvertently compromise the UV stability of materials, requiring a reassessment of the UV stability of plastic compounds that carry the new ‘greener’ additives. However, the present phase of R&D appears to be primarily devoted to identifying substitutes with comparable functionality and increased sustainability. Two classes of additives, both toxic endocrine disruptor chemicals, that can potentially leach out to contaminate the environment [47], deserve special attention.

Of the 10.4 million metric tons (MMT) of plasticisers manufactured worldwide in 2021 [48], about 55% were  phthalates intended for use in poly(vinyl chloride) (PVC), primarily in the building and packaging sectors. Flexible PVC compounds that may carry up to 60 wt% of phthalates account for 80–90% of the consumption. Phthalates, identified as a common pollutant in the environment [49, 50] are also endocrine disruptor (ED) chemicals, that may cause inter-generational adverse impacts on reproductive and neurological health, especially in children [51]. In July 2020, the EU restricted the use of bis (2-ethylhexyl) phthalate (DEHP), benzyl butyl phthalate (BBP), dibutyl phthalate (DBP), and di-isobutyl phthalate (DIBP) in consumer goods or products that posed risks to humans through dermal contact or by inhalation. Likewise, responding to consumer and regulatory pressure, the USA has restricted the use of particular phthalates in plastics.

The validation of sustainable alternatives for phthalates in PVC is well underway [52] and several classes of candidates have been identified: (a) branched and hyperbranched plastics [53–55]; (b) vegetable-oil-based plasticisers [56, 57]; and (c) other promising compounds such as esters derived from biomass [58, 59]. These are being evaluated for their efficacy as plasticisers, especially in PVC, and their migration resistance from moulded plastic products. While selected on the criterion of having effective plasticiser performance, these 'green' substitutes can also affect the UV stability of PVC. Encouraging results were reported for cardanol, a ‘green’ plasticiser for PVC derived from waste cashew nut shells, which also displayed UV-stabiliser properties that are superior to the dioctyl phthalate (DOT) it replaced [60].

Another class of additives being phased out because of their activity as endocrine disruptors and toxicity are flame retardants based on polybrominated diphenyl ether (PBDE) used at 10–20 wt% in some plastic compounds [61–63]. As with plasticisers, the phase-out of PBDEs does not significantly reduce the levels of these compounds in the environment in the medium term, because of persisting residue from their early use since 2004 [64]. PBDE levels in human serum and breast milk are up to 160.3 ng/g [61]. The levels reported in e-waste workers in China are over twice that [65]. The concentration of PBDE replacements in the aquatic environment is higher than that of legacy PBDE’s and these are often bio-accumulative [66]. Exposure to PBDE adversely affects reproductive and neurological health [67, 68]. It is an additive listed in the Stockholm Convention and was phased out in the EU. In the USA, penta- and octa-PBDEs have been banned since 2004 [69], while the decabromo-PBDE, which was excluded, was regulated under the Toxic Substances Control Act in 2021 [70]. China, the largest producer and consumer of fire retardant chemicals, has regulated the levels of penta- and octa-PBDEs, but only in electronic goods that use them in circuit boards [71].

Several ‘greener’ alternatives to PBDEs have emerged, including different (non-PBDE) brominated compounds, organophosphates [72, 73], dechlorane compounds, and aluminium hydroxides [74]. The potential toxicity of the substitute fire retardants is being investigated [75]. The presence of conventional fire retardant additives in the compound often decreases the UV stability of polymers [76]. Whether or not the alternative fire retardants also have this problem has not been fully investigated.

## Wood in building applications

Wood used in building applications, including that exposed routinely to the elements, is expected to be durable over extended periods. Solar UV radiation is well known to cause photodegradation of wood [77–79] as well as of protective paints and coatings used on wood products [80]. Routine exposure of wood to solar UV radiation results in the loss of both their aesthetic and mechanical properties [81]. These changes occur in both natural and artificial weathering, primarily due to photodegradation. Photodamage to wood surfaces is quantified as: (a) changes in surface colour, an important criterion of consumer acceptability of wood [77, 81–83]; and (b) chemical analysis using Fourier transform infrared spectroscopy to assess the extent of oxidative changes [82, 84, 85]. In accelerated weathering of wood [86], a higher intensity of UV radiation and increased temperatures are typically employed, as with plastics; in some instances, a water spray is  also used to simulate the effect of rain [87]. Photodegraded wood contains compounds extracted by water [85] making this a reasonable test parameter.

Cellulose and hemicellulose in wood (unlike its lignin fraction) are structurally saturated compounds carrying no chromophores. It is the absorption of solar UV radiation (290–400 nm) by lignin in wood that initiates the degradation and discolouration often accompanied by surface cracking [77, 78, 88]. Lignin is a strong light absorber with broad absorption in the UV-B (280–315 nm) and UV-A (315–400 nm) wavebands. Higher-density wood species appear to be relatively more stable against UV-induced colour changes [89]. Given the complexity of the mix of products formed, and concurrent biodegradation of the surface, colour changes are only suited for assessing the early stages of weathering [78]. Preferential photodegradation of lignin leaves the wood surface rich in carbohydrate fraction, which encourages biological decay [30]; non-degraded lignin, being phenolic in nature, is more resistant to microbial attack. The rate of photodegradation depends on the anatomical structure of wood species [88]. An exponential dependence of the rate of photodegradation of lignin with temperature was reported in a study based on changes in diffuse reflectance infrared spectroscopy [90]. Surface cracking due to destruction of the middle lamella (that holds adjacent cells together) with a high lignin content, is a major cause of the loss of mechanical properties [81] accompanying the weathering of wood.

Accelerated weathering in the laboratory, where the test conditions can be controlled, results in faster degradation than natural weathering. However, correlating durations of laboratory accelerated exposure with natural weathering remains elusive [91]. The correlation depends on the test parameters employed [92].

### Thermal treatment for improved resistance to UV radiation

Heat-treated wood is a sustainable alternative to pressure-impregnated wood that is resistant to UV-induced and biological degradation. Environmental considerations have made the chemical-free heat treatment of wood an increasingly popular approach to increase durability. Subjecting wood to high temperatures under humid conditions in heat treatment, results primarily in the cleavage of the acetyl groups of the hemicellulose fraction and its hydrolysis into oligomeric products. Thermal treatment yields appealingly darker [93, 94] treated wood that is less hygroscopic and has improved dimensional stability [95]. Neural network-based algorithms that predict the properties of treated wood as a function of variables such as temperature, are being developed [96]. However, these improvements are obtained at the expense of its UV resistance with the treated wood discolouring readily on exposure to solar UV radiation [97, 98]. Coating the treated surface can address this limitation. However, designing chemically coatings that adhere well to the hydrophobic surface of treated wood is a challenge, but several promising candidates have been identified.

Impregnating Scots pine wood with a titania sol in paraffin (at 212 °C) improved its UV resistance [98], with the colour change and lignin degradation reduced by more than 50% with the treatment compared to the control, after 1176 h of accelerated weathering. Ashwood heat-treated at 192–212 °C and clear-coated with polyurethane coating also yielded good resistance to discolouration in laboratory exposure of up to 2000 h under UV-340 fluorescent lamps [99]. Further treatment of hardened surfaces with air saturated with steam at 95–135 °C enhances the colour stability of wood. For instance, change in surface colour after 298 h exposure to a xenon Weather-O-meter (simulates solar radiation with filtering systems designed for weathering) of untreated maple wood was reduced by 31.8, 43.8, and 61.1% with steam treatment at 95, 115, and 135 °C, respectively [100]. While thermal treatment improves the desirable characteristics of wood, the accompanying reduced outdoor lifetimes under solar UV irradiation needs to be controlled using cost-effective surface coatings for wider acceptance of the approach.

### Sustainable stabilisers derived from wood

As with plastics, sustainability considerations drive the search for non-toxic sustainable UV stabilisers for protecting the wood in outdoor applications. Plant-based compounds have shown promise as efficient UV stabilisers controlling surface discolouration in a range of wood species [101–105]. Flavonoids extracted from *Acacia confusa* heartwood significantly reduced the UV-induced degradation of lignin in wood [101]. Two most abundant flavones (okanin and melanoxetin) in *Acacia confu*s*a* sp., have marked radical-scavenging and singlet-oxygen quenching properties [102]. Extractives of the wood of other species, such as that of Japanese cedar (*Cryptomeria japonica*) [106], Merbau (*Intsia* sp.) [31, 107] *Dalbergia cohinchinensis* [31] as well as the bark of Trembling Aspen (*Populus tremuloides*), lodgepole pine (*Pinus contorta*), and western red cedar (*Thuja plicata*) [108] have all been demonstrated to have UV-stabilising activity either in wood or wood composites.

Wood bark extracts are particularly rich in polyphenols including condensed tannins and flavonoids, which are very good absorbers of UV radiation. For instance, a 1 wt% solution of the extractive of *Phoebe zhennan* wood by polar solvent ethyl acetate completely absorbs solar UV-A and UV-B wavelengths in laboratory studies [109]. The extractives accounted for 3.8 wt% of the wood. Such extracts can be used to stabilise surface coatings on wood as well. Polyurethane-acrylate wood coatings stabilised with 2 wt% of the bark extracts from Chinese fir, for instance, effectively controlled the photo-discolouration of wood [108]. The colour change on exposure to a UV source for 898 h was reduced by *ca* 67% compared to a control coated with polyurethane without any extractive. Also, extracts from the bark of alder and maritime pine species at a concentration of 5% in alkyd-based coatings provided excellent UV protection of Scot pine surfaces [110]. These stabilisers also work well in WPCs that also undergo UV-induced discolouration and loss of mechanical integrity [111]. Bark extractives from Western red cedar (*Thuja plicata*) at 2 wt% mixed into bulk wood-plastic showed good stabiliser effectiveness, yielding about 25% less discolouration at 1200 h of accelerated weathering, with less severe surface cracking and chemical degradation as indicated by Fourier transform infrared spectroscopy [1].

Lignin is the second most abundant biopolymer after cellulose in wood and is rich in UV-absorbing chromophores. It can therefore serve as a promising ‘greener’ substitute for synthetic UV absorbers [112, 113]. The UV-shielding properties of lignin depend upon its extraction process, methoxyl content, and the shape and size of lignin domains. Lignin NPs have even better UV-shielding properties. However, the undesirable dark colour of lignin is a drawback with lignin-based UV-absorbing products. Laboratory exposures of coatings with lignin NPs derived from waste wood on beech wood panels, show good control of UV-induced discolouration [114].

### Novel wood-based building materials

**Fig. 2:**
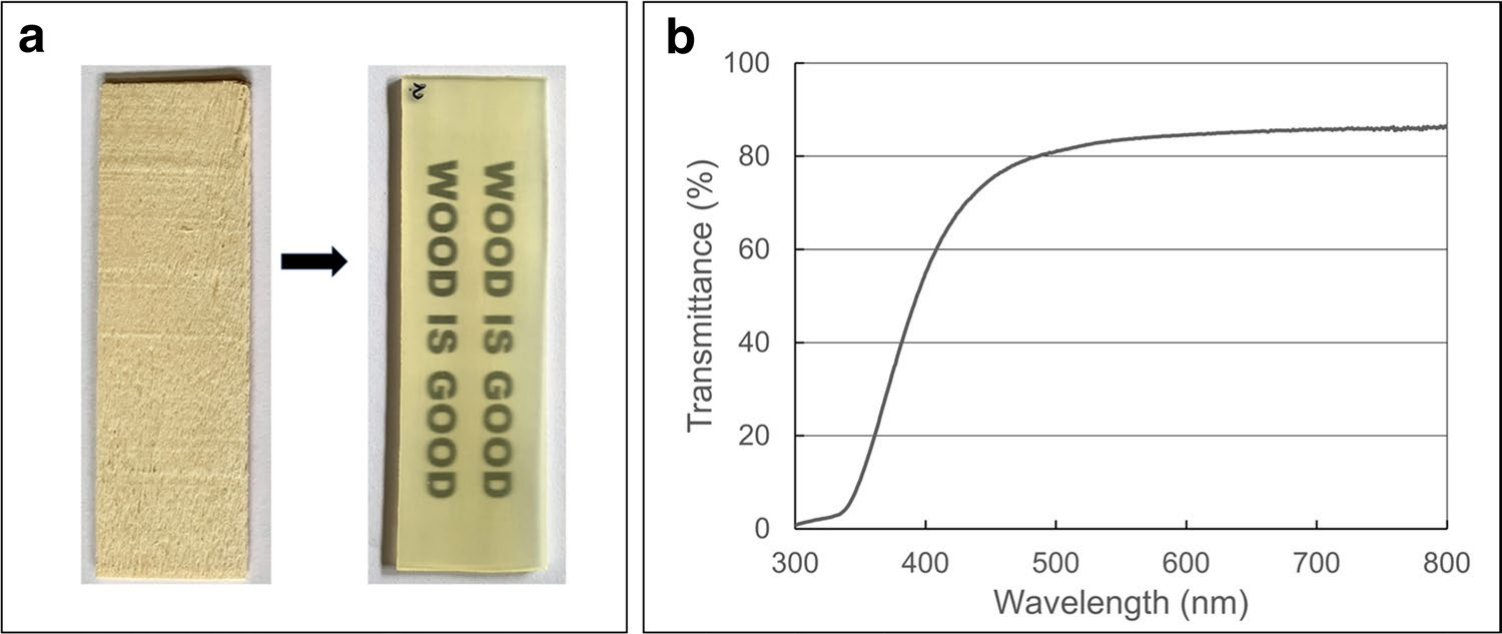
**a** Piece of poplar wood (left) and piece of transparent wood composite (TWC, right) prepared using poplar wood and epoxy resin; **b** optical transmittance of TWC of 2 mm thickness

A novel, scalable, and optically transparent UV-blocking wood composite is a potential sustainable replacement for glazing (plastics or glass) in buildings and in greenhouse applications. Some of these novel transparent wood composite (TWC) materials have better UV stability compared to polycarbonates used in glazing. In this technology, the lignin fraction of wood is replaced by either a synthetic [115] or a biopolymer [116, 117], to obtain a composite of cellulosic materials and polymer (Fig. 2). For instance, a 2 mm thick sample of Douglas fir wood/epoxy TWC, transmits 80% of visible light but blocks UV radiation over the wavelength range of 200–400 nm [115]. A luminescent TWC was reported from Basswood with poly(methyl methacrylate) (PMMA) [118]. The synthetic fraction included the additives, ammonium polyphosphate (APP), and lanthanide-doped strontium aluminate (LSA; SrAl2O4:Eu^2+^, Dy^3+^) phosphor nanoparticles. The luminescent material is suited for applications such as smart windows, lighting, and safety directional signs in buildings. With good thermal stability up to 315 °C, TWCs can be designed to filter out UV-B radiation and have potential applications in smart building technology [119, 120]. TWC materials based on biopolymers such as chitosan or cellulose in place of synthetic resins, have also been synthesised [116]. For instance the use of cellulose–lignin slurry from poplar wood displays high mechanical strength, excellent water stability, resistance to UV radiation, and improved thermal stability [121].

However, even with the chromophore-carrying lignin removed, TWCs still undergo some photo-discolouration and degradation on extended exposure to solar UV radiation [122, 123] that might be due to photodegradation of residual lignin or the polymeric component (epoxy in this case). With extended exposure to solar UV radiation, the transparency of the material is compromised. This drawback, however, can be addressed using established UV-stabiliser technologies. Blending 1.0 wt% of a conventional UV stabiliser (2-(2H-benzotriazol-2-yl)-4, 6-di-tert-pentylphenol) into the epoxy resin used in epoxy/poplar wood TWC, reduced the loss in transmittance of visible light on weathering. For example, after 250 h of irradiation under UVA-340 fluorescent lamps, only a 1.4% loss in transmittance was obtained with the stabilised TWC at 550 nm compared to a 27.5% decrease in the untreated controls [122].

## Nanoparticulate filler-UV stabilisers

### Nano-oxides and aramid fibres

Aramids constitute a high-value, high-performance class of industrial fibres that account for a few percent of the global synthetic fibre production. These fibres with exceptionally high strength (2.4–3 GPa) and modulus of elasticity (45–160 GPa) [124] are well suited for demanding applications such as bullet-proof vests, firefighter protective garments, and in military applications [125–127]. Nanofibres of aramids in the size range of 10–100 nm are also used to reinforce nanocomposites [128]. However, a significant drawback of the material is their inherent poor UV stability, mostly because of their aromatic structural features, limiting their use in outdoor applications [129]. It is challenging to incorporate UV stabilisers into aramids as organic compounds do not dissolve well in the highly crystalline fibre. Nano-fillers of wide-band-gap oxides, which decorate the fibre surface or are grown on the fibre's surface [130], are especially effective stabilisers. Different strategies based on nanoscale oxide fillers are being developed to improve the UV stability of aramids.

**Fig. 3:**
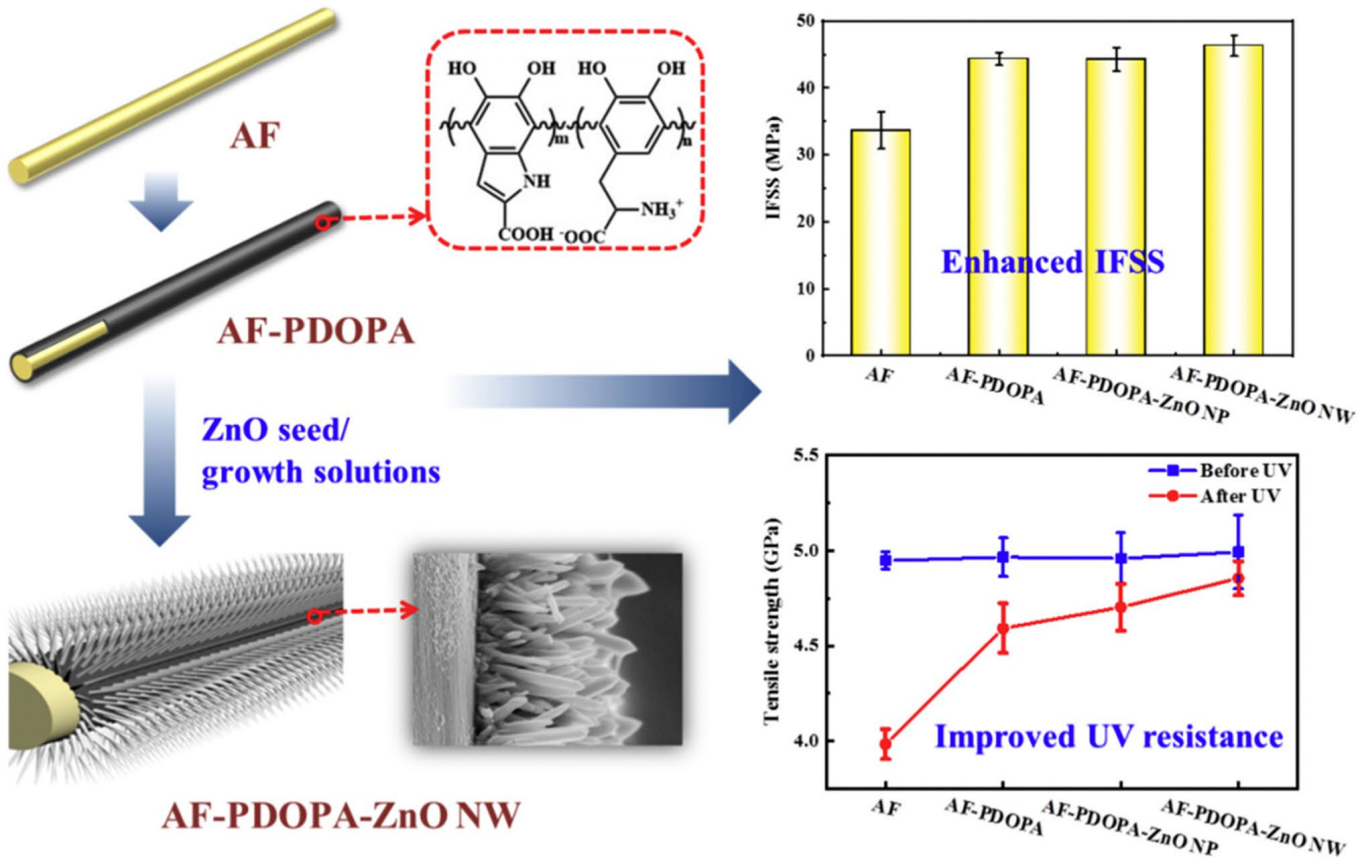
Seeding of ZnO nanoparticles onto aramid fibres (AF) and subsequent growing of ZnO nanowires (NW) on the seeded layer for improved UV resistance and enhanced mechanical properties of the fibres; PDOPA refers to poly l-3,4-dihydroxyphenylalanine, IFSS to interfacial shear strength. Figure from [131], CC BY NC ND

Ma et al. [130] functionalised aramid fibres with nano-ZnO-structures grown on the fibre surface, resulting in improved UV resistance. After 168 h of UV irradiation under a 3-kW source with a wavelength range of 290–365 nm, the loss in tensile strength of the surface-modified was less than 5%, while it was 20% in the unmodified fibre. Seeding ZnO NPs onto aramid fibre surfaces coated with poly L-3,4-dihydroxyphenylalanine resulted in ZnO nanowires growing in the seeded layer as illustrated in Fig. 3 [131]. After 168 h of UV irradiation of 0.76 W m^−2^ nm^−1^ at 340 nm, the retention of the tensile strength for the oxide-modified and control fibres was 97.2 and 80.4%, respectively. ZnO NPs have also been crystallised onto aramid fibres by a sol–gel process with drying in supercritical CO_2_. Subsequent exposure to UV radiation for up to 216 h, under a 40 W UV lamp (wavelength range 280–315 nm, complying with the Chinese Standard GB/T 14522-93) at 60 °C, the treated aramid fibres retained 93% of their average tensile strength, while that of control fibres was only ca 25% [132]. While the quantitative results of these studies are not comparable due to differences in the experimental settings, they indicate the potential of these techniques in improving the performance of aramid fibres in a way that could be transferable to functionalisation of other materials.

Sun et al. [129] used drying in supercritical CO_2_ to improve the UV resistance and durability of aramid fibres treated with TiO_2_ NPs. When exposed for 168 h under a UV-B lamp (40 W, wavelength range 280–315 nm, lamp length of 1220 mm, (Dongguan Instrument Co. Ltd., Guangzhou, China), yielding an exposure of 40 W m^−2^ complying with the Chinese Standard GB/T 14522-93), absorption of UV-B radiation increased by ~ 15% at a TiO_2_ loading of 2.38 wt% on the fibre surface. The tensile strength and modulus retention also improved by 5–14%. Surface decoration of fibres with nano-oxides suggests a useful approach to improving the UV stability of other fibres. Where supercritical CO_2_ is used, the technique needs to be evaluated for economic feasibility.

### Nanocomposite films

Nanoparticle (NP) additives increasingly find application as UV stabilisers in films, as demonstrated by their use in greenhouse covers; for instance, 2 wt% each (total 6 wt%) of ZnO, TiO_2_, and SiO_2_ NPs in a size range of 20–45 nm as light stabilisers, has been incorporated into PET films [133]. The mixture of three NPs improved the UV-blocking properties (94% under UV-B and 60% under UV-A radiation). A reduction of 57% in UV-B and 36% in UV-A transmission relative to that by a control film was achieved without affecting the high photosynthetically active radiation (PAR, 400–700 nm) transmittance of the film. Moreover, these properties persisted over 90 days of irradiation under a Xe lamp (Air Mass 1.5 Standard Spectrum, 60 W m^−2^). Functionalised NPs, such as those coated to obtain a double-shell structure, are sophisticated nanofillers with tailored properties. ZnO NPs coated with amphiphilic polyurethane (APU) was investigated as a stabiliser in UV-cured poly(urethane acrylate) (PUA) [134]. Transparent polymer films with NP loading of 0.3 wt% exhibited a  >50% increase in tensile strength and up to 80% absorbance of UV-B radiation compared to the untreated PUA. The APU-functionalised ZnOs are effective multifunctional fillers even at the low concentrations used in these studies. TiO_2_ NPs have been coated with SiO_2_ and then poly(D-lactide) (PDLA) grafted onto the coated surface to obtain a double-shell (TiO_2_@SiO2-g-PDLA) hybrid nanoparticle [135]. When incorporated into a poly(l-lactide) (PLLA) matrix (at a loading of 2 wt% TiO_2_), the hybrid nanocomposite showed almost a tenfold increase in UV absorbance at 350 nm relative to the untreated PLLA and a higher tensile strength, even after 72 h irradiation under a 300 W UV lamp. Sophisticated core–shell or double-core–shell NPs used in these studies may be expensive relative to NP-fillers. The fact that they are used at such low weight fractions to obtain desirable multiple functionalities may offset their cost at least in some film applications.

### Nanoscale carbon fillers in plastics

Carbon materials are excellent absorbers of both visible and UV radiation. They may also be used as reinforcing fillers in polymer composites. [136]. In addition to reinforcement, fillers like carbon nanotubes (CNTs) and graphene-family nanomaterials (GFNs) also impart increased UV stability to polymer composites. GFNs and CNTs are used, for instance, in batteries, sensors, and biomedical (antibacterial) applications. [137, 138]. Because of their small dimensions, nanomaterials are very efficient in UV-shielding photostabilisation.

UV-resistant glass-fibre-reinforced composites have been produced by dispersing 0.25–1.0 wt% of multi-walled carbon nanotubes in fibre-filled epoxy matrices [139]. When exposed under a UV radiation source (six spectral UVA-365 lamps with nominal wavelength 365 nm, Worldwide Specialty Lamp, Austell, GA), yielding 0.77–0.95 W m^−2^ nm^−1^ to comply with the ASTM specification of 0.89 W m^−2^ nm^−1^, for 2160 h, in cycles of exposure (4 h light and 4 h darkness) the filled composites showed very good UV resistance as evidenced by the absence of microcracks typically observed in untreated composite samples. Epoxy nanocomposites that contain 0.5 wt% multiwalled carbon nanotubes (MWCNTs) (outer/inner diameter < 20 nm/4 nm, length 1–12 μm) dispersed in the resin that was based on diglycidyl ether of bisphenol A (DGEBA) and cured with 2,2,4-trimethylene-1,6-hexadiamine (TMDA) [140] have been studied. These were subjected to accelerated weathering under UV-A lamps (140 W m^−2^ over 295–400 nm range) in alternating humidity and temperature conditions for a total irradiation time of 1–6 months. The DGEBA–TMDA/0.5 wt% MWCNT samples displayed improved resistance against UV degradation compared to the DGEBA–TMDA samples with no carbon filler. A novel UV stabiliser for polypropylene has also been synthesised, based on functionalised graphene oxide (GO, thickness 3.4–7 nm, diameter 10–50 μm), where a hindered amine stabiliser was grafted onto GO nanoplatelets [141]. Samples of the plastic stabilised with 0.5 wt% of GO and functionalised GO were artificially aged under OSRAM Ultra Vitalux 300 W sunlamp up to 600 h. The functionalised filler efficiently retarded the rate of photo-oxidation, as measured by the retention of tensile properties. Hybrids of graphene (8 nm flakes) and MWCNTs (length 5–15 μm, diameter 10–30 nm) have been used in waterborne polymer coating synthesised via mini-emulsion polymerisation [142]. Only 1 wt% of hybrid G/CNT was used in the 50 wt% MMA and 50 wt% butyl acrylate (BA) resin. This was adequate to completely suppress photodegradation in accelerated ageing of 400 h under a UV lamp (nominal wavelength 366 nm, P-Lab, 550 mW cm^−2^, distance 15 cm) at 55 °C [142].

Studies reporting on the enhanced performance of materials with nanoscale carbon fillers are specific in terms of the size of the filler, the technique applied, the polymers and fillers used, the ageing conditions and the characterisation methods employed. However, it would seem that these kinds of fillers are finding more applications as the demand for more durable and more sustainable materials increases. In these recent studies it is significant that less than 1 wt% of the carbonaceous filler was able to impart very significant improvements in UV stability of the thermoset composites studied.

Graphene is currently used in electronics as well as in composites to take advantage of its unique electrical and reinforcing characteristics. The accompanying photostabilisation can be important in composite applications. High-volume use of graphene in composites is currently limited by the high manufacturing costs and uncertainties related to their lifetime, operational stability, and reproducibility [143]. In the future, graphene is nevertheless expected to find ever more applications (e.g. sensors, solar cells, and self-cleaning surfaces) [144–146].

## Photovoltaic module components

Solar photovoltaic (PV) energy is a leading sustainable substitute for fossil-fuel energy and its rapid installation is critical to meeting the agreed-upon reductions in future global CO_2_ emissions [5]. This will require the continuation of the accelerated growth of the PV industry globally. In 2020, there was ~ 940 gigawatt peak (GWp) of installed capacity. The newly installed 173 GWp in 2021 was the largest ever installed capacity in a single year. The continued competitiveness of PV power generation will be supported by the reduction in PV system costs by optimising manufacturing and having increased cell and module performance. The market is still dominated by crystalline silicon modules, but has shifted to mono-silicon cells with new cell types being commercialised. Bifacial modules have been introduced in the market. The production capacity of PV has increased to over 470 GWp with the expansion of larger wafers and improved module area efficiency. Modules have grown in efficiency with passivated emitter and rear contact (PERC) module and half-cell interconnect technologies. Maintaining long service lifetimes with minimal failure rates for PV modules is essential to meet the ambitious rates of growth expected of the technology [147]. Given the recent large-scale installations of PV modules and their long lifetimes (warranties of 25–30 years on performance), accelerated laboratory testing is also used in addition to field data to identify potential degradation mechanisms and failure risks. Accelerated laboratory testing has been changing to better understand the different degradation mechanisms that are present in PV modules; however, these types of testing do not always provide accurate prediction of degradation under in-use conditions and correlation between accelerated testing and in-use conditions is still lacking [148, 149].

**Fig. 4:**
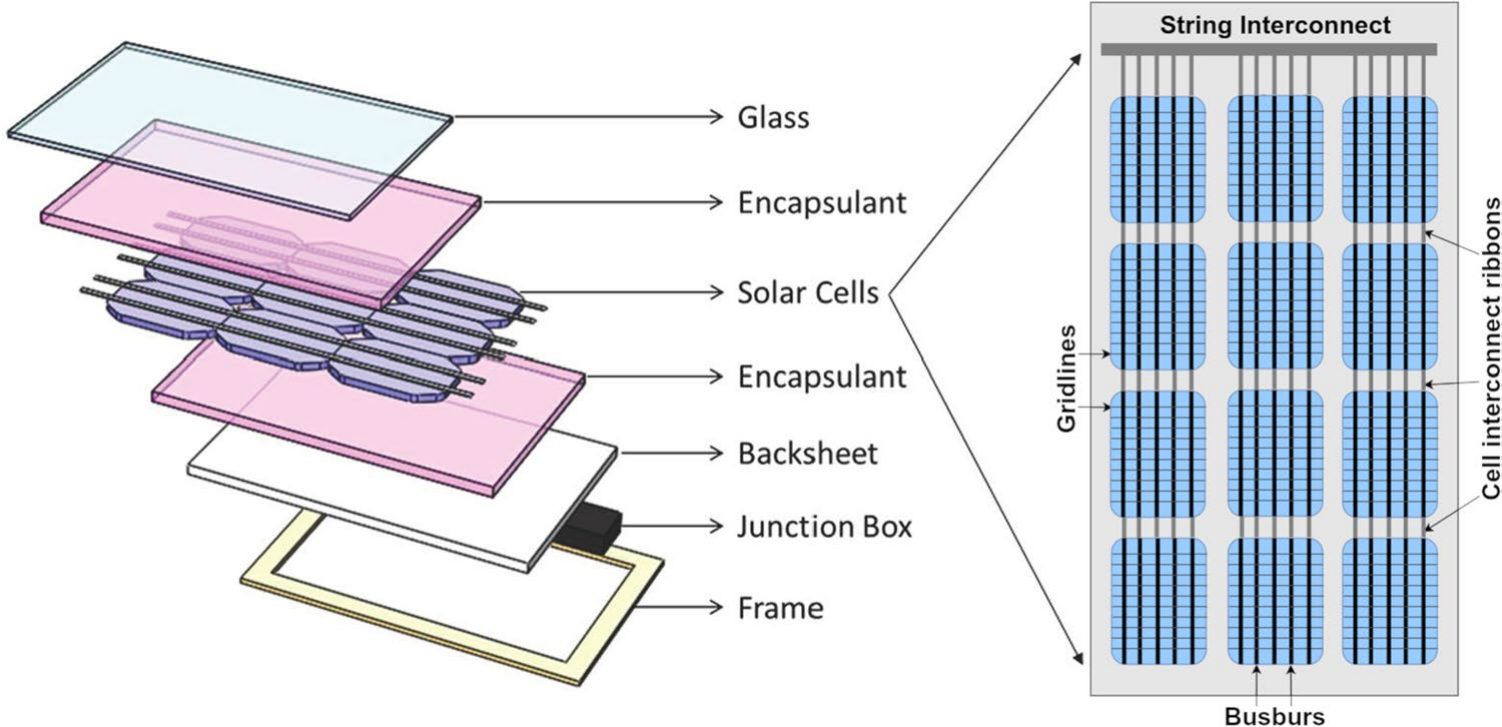
Components of a crystalline silicon photovoltaic (PV) module illustrating the placement of encapsulants and backsheet, both made of plastic materials prone to degradation by solar UV radiation (modified from [187])

**Fig. 5:**
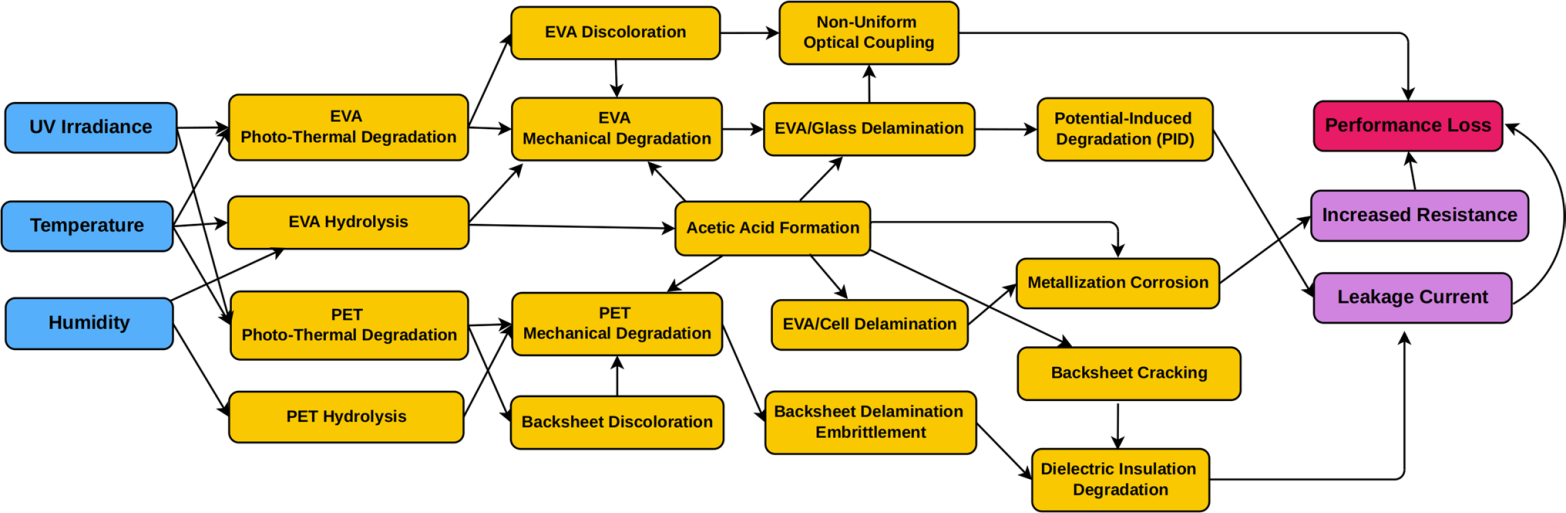
An example of potential degradation pathways in a photovoltaic (PV) module showing that there are synergistic effects from different stressors (blue) including ultraviolet (UV) radiation that lead to degradation mechanisms and mode (orange). This degradation then causes overall performance loss (purple, red) such as power loss in PV modules (modified from [187]). PET, poly(ethylene terephthalate); EVA, ethylene vinyl acetate

The basic structure of a crystalline silicon PV module is shown in Fig. 4. The encapsulant is a protective adhesive layer of a soft polymer that is used to cover the top and the bottom of the active layer (the solar cells). The encapsulant provides electrical insulation and physical support for the fragile cells and has historically been made of ethylene vinyl acetate (EVA) copolymers. A second plastic component is the backsheet, which provides environmental protection for the module and safety for humans to the high voltage. It is typically made of three polymer layers (outer or air-side, core, and inner layer construction) with the inner layer typically consisting of PET. In the majority of backsheets, the multiple layers are combined with an adhesive layer while some backsheets are coextruded [150]. Encapsulant and backsheet materials are key to ensuring PV module lifetime and are major components of the cost of the module. Degradation in each component layer in a PV module can accelerate degradation in other component layers (Fig. 5).

Transparent EVA is the most common encapsulant and will continue to be the most widely used encapsulant (~ 52% in the next 10 years). Additionally, EVA with TiO_2_ filler (white) will claim ~ 10% of the market share to be used with bifacial cells. Polyolefin is expected to be 20% and extruded EVA with polyolefin ~ 10% in the next 10 years [147]. Polymer backsheets will continue to be the most common materials, with PET core-layers continuing to be used to provide electrical resistance, and with the outer layer of poly(vinylidene difluoride) (PVDF) as the main backsheet type. However, in the next 10 years polymer backsheets are expected to decrease from ~ 75 to 45% of the market share with glass–glass modules increasing over that time [147]. Solar UV radiation-induced degradation of these plastic components is a major factor in PV module failure [151] and many material failures in PV modules observed in the field involve encapsulant degradation, backsheet failure, or de-bonding of elements due to adhesive failure [151–154].

### Durable encapsulation strategies

It is critical to select encapsulants that are durable under continuous outdoor exposure; discolouration of encapsulants accounts for about 10% of the module failure [153]. Transparent EVA is the most common encapsulant in PV modules [147]. Still, there is a need for a more durable, UV-stable material for next-generation encapsulants. Degradation of EVA encapsulant appears to be a complex inhomogeneous process involving solar UV radiation, temperature, and moisture. The installation location and how the modules are installed, especially their tilt angle (e.g. normal through 90°) can affect the amount of UV irradiance the module is exposed to [155]. The overall microclimate or stressor conditions within a PV module are non-uniform due to differences in moisture ingress, oxygen diffusion, module temperature, and physical strain within the module. There is a relationship between the photodegradation of the frontside EVA encapsulant and the location of the EVA in the module. Oxygen and moisture diffuse through the backsheet and the edges of the module; therefore, frontside EVA has lower transmittance at the centre of the cell compared to the edges of the cells. This is due to oxygen bleaching of photodegradation produced chromophores [156]. EVA is impacted on the edges of the solar cells more so than other locations, resulting in the formation of acetic acid, which leads to corrosion of metallisation and power loss [157]. A range of EVAs with different copolymer compositions are available commercially. Based on an accelerated weathering study of several candidate EVA copolymers, one with 18–33% by weight of vinyl acetate was found to be the most UV stable, making it potentially well suited for PV use [158].

The ongoing effort to find a better replacement for the encapsulant material has yielded several candidate polymers, such as ionomers and thermoplastic polyolefin (TPO) [159]. TPO has been investigated as an alternative to EVA [160, 161] and has superior thermal stability, adhesive strength and resistance to discolouration. For instance, the discolouration of TPO on exposure to UV radiation of 365 nm from an LED light source at 900 W m^−2^ at 90 °C and 10–13% RH was found to be nine times slower relative to EVA, and the polymer may also be easier to process [160]. While the initial research on TPO is promising, developmental work to validate its efficacy and economic feasibility is necessary especially in field studies of operational PV modules [162].

Among the candidate polymers being investigated is thermoplastic polyurethane (TPU). A multi-laboratory study compared five representative formulations of EVA encapsulants to TPU encapsulants of unknown formulation. Different sources including xenon, UVA‐340, and metal‐halide lamps with different filter types were used in the study. Samples were continuously exposed for 180 days at a relative humidity (RH) ranging from 7 to 50% and temperatures from 40 to 90 °C. The encapsulants, EVA and TPU, exposed in the dark conditions showed minimal degradation compared to those exposed to UV radiation, which drives degradation. UV radiation-induced degradation was synergistically impacted at higher temperatures and humidity over the duration of exposure. The TPU in this particular study had the largest decrease in transmittance compared to the EVA formulations. The variability in degradation behaviour of the five EVAs suggests that their copolymer composition, residual monomer, and additives are important determinants of their UV degradation behaviour [163]. The synergy between UV radiation and temperature identifies climate change as a possible risk factor in reducing the service lifetime of these modules.

The UV degradation of four different encapsulants, TPO, TPU, silicone, and EVA was determined under accelerated ageing while laminated between glass. A 1000 W Hg-Xe arc lamp was used with an accelerated exposure over 1694 h. This was calculated to be equivalent to 2.74 years of natural exposure to UV radiation in Atacama Desert conditions, corresponding to 1.34 years of UV-A and 24.7 years of UV-B exposure [164]. The TPU and silicone candidate materials yielded a 5 and 0.9% loss in the transmittance in visible light, respectively. TPO and EVA underwent similar chemical changes determined by Raman spectroscopy*,* but EVA showed a slightly lower reduction in light transmittance [164]. One study suggests that the more UV-transparent the encapsulant is, the higher the short circuit (*I*_sc_) losses in heterojunction (HJT) PV cells will be, which is not the case with other architectures [165]. Although silicon HJT cells are presently a small fraction of the commercial PV market, they are currently being considered as the next step in technology because of their ability to respond to a broader spectrum compared to current PERC technology [147]. This suggests that UV radiation through the encapsulant has different degradation on the cell technology.

### Durable backsheets

The most common types of backsheet have an outer layer made of either PVDF, PET, polyvinyl fluoride (PVF), polyamide (PA), or fluoroethylene vinyl ether (FEVE). Additional research on new types of backsheets is underway [166]. These polymeric backsheets can fail if they crack due to environmental degradation, leading to potential electrical leakage, causing PV modules to be replaced or repaired to address the safety concern [154]. Repairing defects in PV module backsheets in the field has become a new approach in order to prevent the need to fully replace existing installed modules [167, 168]. Backsheets are exposed to UV irradiance from the front side of the PV module in between the PV cells and from the rear-side albedo. White backsheets often turn yellow due to UV degradation and although these backsheets do not have an optical function, yellowing can cause an aesthetic problem especially in PV modules mounted in car parks. Importantly, yellowing is an indicator of degradation in progress of the backsheet, which can lead to more severe degradation. Advanced stages of degradation lead to delamination, embrittlement, or cracking resulting in the backsheet no longer providing electrical insulation [154, 169–171]. Cross-sections of weathered-adhered laminate sandwich constructions of a PV module (glass/encapsulate/backsheet), using spatially resolved fluorescence imaging, for instance, show disproportionately high UV radiation damage to the adhesive layers [170].

The drive to reduce cost in PV modules is one reason for the use of materials of relatively lower UV stability in PV modules. Backsheet design would benefit from inherently more UV-stable outer layers, such as fluoropolymers. The addition of UV stabilisers in these long-lived materials is problematic due to stabiliser loss by leaching and bleaching over the greater than 25-year lifetime [172]. However, many backsheets are now coextruded, which removes the need for adhesive layers between the three layers.

The backsheets installed in PV modules in commercial fields had non-uniform degradation at the edges of racks/rows due to the increased UV albedo from the ground that is reflected on the backsheets that are less shaded than the interior of the modules [173–176]. The ground cover under the backsheets influences the amount of reflected UV radiation on the backsheet. For example, grass has a lower albedo than white rock [173, 175] and leads to less degradation by UV radiation on the backsheet. The mounting configuration and module design could be improved to reduce the effect of the UV albedo on PV module backsheets [173, 175, 177]. The rear-side albedo (visible irradiance) helps increase power production in bifacial PV modules and sites would be designed to maximise albedo. This needs to be considered in bifacial modules with transparent polymeric backsheets [174].

The stability and insulation efficiency of the backsheet are related to the choice of encapsulant in the module. UV-induced degradation coupled with hydrolysis of EVA can produce acetic acid that can affect the backsheet, as illustrated by the crack formation in polyamide backsheets [170, 178–180]. EVA copolymer is sometimes used as an inner layer in multi-layer backsheet construction. The EVA inner layer on exposure to UV radiation causes polyamide to crack [178, 179]. In these complex systems, it is very important to study the interfaces between layers because degradation in one component can initiate additional degradation in other components. This means that accelerated testing needs to account for the synergistic impacts of the system and changes to materials in a PV module can impact the lifetime of other materials. Currently ~ 12 GW of installed modules with polyamide backsheets have failed due to cracking, which has a large impact on the installed PV power plants [180].

Backsheets with a PET outer layer readily undergo degradation under exposure to UV radiation as demonstrated in a study conducted in the NIST Simulated Photodegradation via High Energy Radiant Exposure (SPHERE) weathering chamber (170 W m^−2^ irradiation with a wavelength range of 290–400 nm) for 1800 h [181]. However, this surface degradation has not been shown to impact electrical output of the module. Upon UV degradation, (PET)/PET/EVA backsheets developed surface cracks (in the white TiO_2_ filled PET outer/air-side layer) [182]. Cracking was absent in samples aged without UV irradiation. Moisture and UV irradiance appear to have synergistic roles with UV radiation initiating the surface cracking, while moisture enhances the surface degradation, decreasing the fracture toughness [182].

Novel transparent backsheets designed to work specifically with bifacial PV cells and modules are being introduced. These modules will collect the reflected albedo light on the backside of the module to increase power generation per unit area. Bifacial modules have been designed with the back surface metalised. Three types of transparent backsheets (outer/core/inner) have been tested including a PVF/PET/FEVE_1_ coating backsheet, PVDF/PET/FEVE_2_, and a FEVE_3_/PET/EVA [183]. The backsheets and laminates (glass, encapsulant, transparent backsheet) were exposed to UV irradiance in the NIST SPHERE under *ca* 140 W m^−2^ (295–400 nm) at 75 °C/50% RH (transparent films) and 65 °C/50% RH (laminates). For all three backsheets, the PET core layer demonstrated the greatest material property changes after UV irradiation. This layer is the most susceptible to degradation by UV radiation. After exposure to the radiation, the outer layer of the fluoropolymer showed an increase in modulus and hardness, indicating this layer had become brittle due to degradation. The FEVE_3_/PET/EVA showed the most deterioration in optical, chemical, mechanical, and thermal properties after exposure, which eventually led to cracks within layers of the PET core, adhesive, and EVA inner layers, while the FEVE outer layer largely remained intact.

### Cable sheath

Contributing to electrical power loss in PV systems and in electricity transport is the degradation of cable sheaths, which cause power loss and safety concerns. These undergo oxidation under solar UV irradiation causing brittleness [184, 185]. For instance, crosslinked polyethylene (XLPE) material, widely used for manufacturing high-voltage cables as an electrical insulation material, was exposed to 36 W low-pressure vapour fluorescent lamps with radiation ranging from 350 to 400 nm for 200 h, resulting in a change in colour from grey to dark yellow following the exposure [186]. Photo-oxidation results in polymer chain breakage and the formation of unsaturated groups, such as vinylidene and vinyl groups in PE. After weathering exposure, the surface of the XLPE insulating material is rough and degraded. The surface becomes non-uniform, since the material is semicrystalline and photo-oxidation occurs preferentially in the amorphous region. These surface changes from laboratory-based exposure lead to fatigue in the XLPE surface resulting in breakage in the insulating material [186].

## Micro- and nanoparticle and composite fibres

Nanoparticles are used to coat or decorate the surface of textile fibres to impart specialised properties to the fibre and fabric, such as antibacterial action using silver particles [188, 189], wound healing [190], and solar energy harvesting [191]. When the NPs are also UV-absorbing or scattering, these fabrics display incidental UV-shielding to protect wearers from solar UV radiation. For instance, textile fibres surface-coated with silver NPs for antibacterial performance also provide a high degree of UV-screening because of the opacity of the particles [192]. However, the decorating NPs can also be selected primarily for their UV-screening to develop effective fabrics. The approach is not entirely novel, but the use of well-defined nanoparticles provide impressive UV-shielding (Table 1). The UV protection factor (UPF) quantifies the efficiency of the fabric, and would also depend on weave characteristics; a UPF of x would mean that only 1/*x* of the solar UV-A and UV-B radiation would be transmitted through the fabric.

**Table 1:** Nanoparticles used as surface treatment on textile fibres to increase their UV-shielding

Materials	Particles			UPF (max)	Comments	References
	Type	Size	Amount			
Cotton fabric	TiO_2_	17.9 nm (crystal-lite)	2.61% Atomic Ti	220	Maintained appreciable UPF after 15–20 washing cycles	[199]
Cotton fabric	TiO_2_	90–150 nm	Treated with 1% wt./v TiO_2_ NPs sol	277	UPF reduced by 64.9% after 15 wash cycles	[197]
PVDF film	TiO_2_	TiO_2_ pigment	10.55% Atomic Ti	Only 0.01% UV transmittance in the entire spectrum	Less strength loss after natural weathering for 30 days	[198]
Poly(ethylene oxide) (PEO) nanofibre	TiO_2_	100 nm	3.81% Atomic Ti	2751.65	Transmittance values of UV‐A & UV‐B reduced to 0.0525% and 0.0207%	[200]
Cotton fabric	ZnO	20 nm (crystallite); 80–85 nm (agglom-erated)	Treated with 2% ZnO NPs sol	58	Significantly enhanced self-cleaning/stain removal properties	[201]
Cotton fabric	ZnO	38 nm	22.8% Atomic Zn	102.88	Little decrease in UPF after 20 washing cycles	[202]
Cotton fabric	ZnO	35–42 nm (crystallite), 2405 nm (aggregate)	Treated with 20 mM Zn^2+^ precursor sol	> 96% blocking in UV-A and UV-B wavebands	Good efficiency in methylene blue (MB) degradation, self-cleaning properties	[203]
Cotton fabric	Ag	35–80 nm	Treated with 4 wt% silver carbamate sol	2396	Improved photochemical degradation of MB under UV radiation	[204]
Cotton fabric	MgO	9 nm (crystallite); 3040 nm	Treated with 2% MgO NPs sol	71	Significantly enhanced self-cleaning/stain removal properties	[201]

### UV-protection fabrics based on nano-oxides in textile fibres

**Fig. 6:**
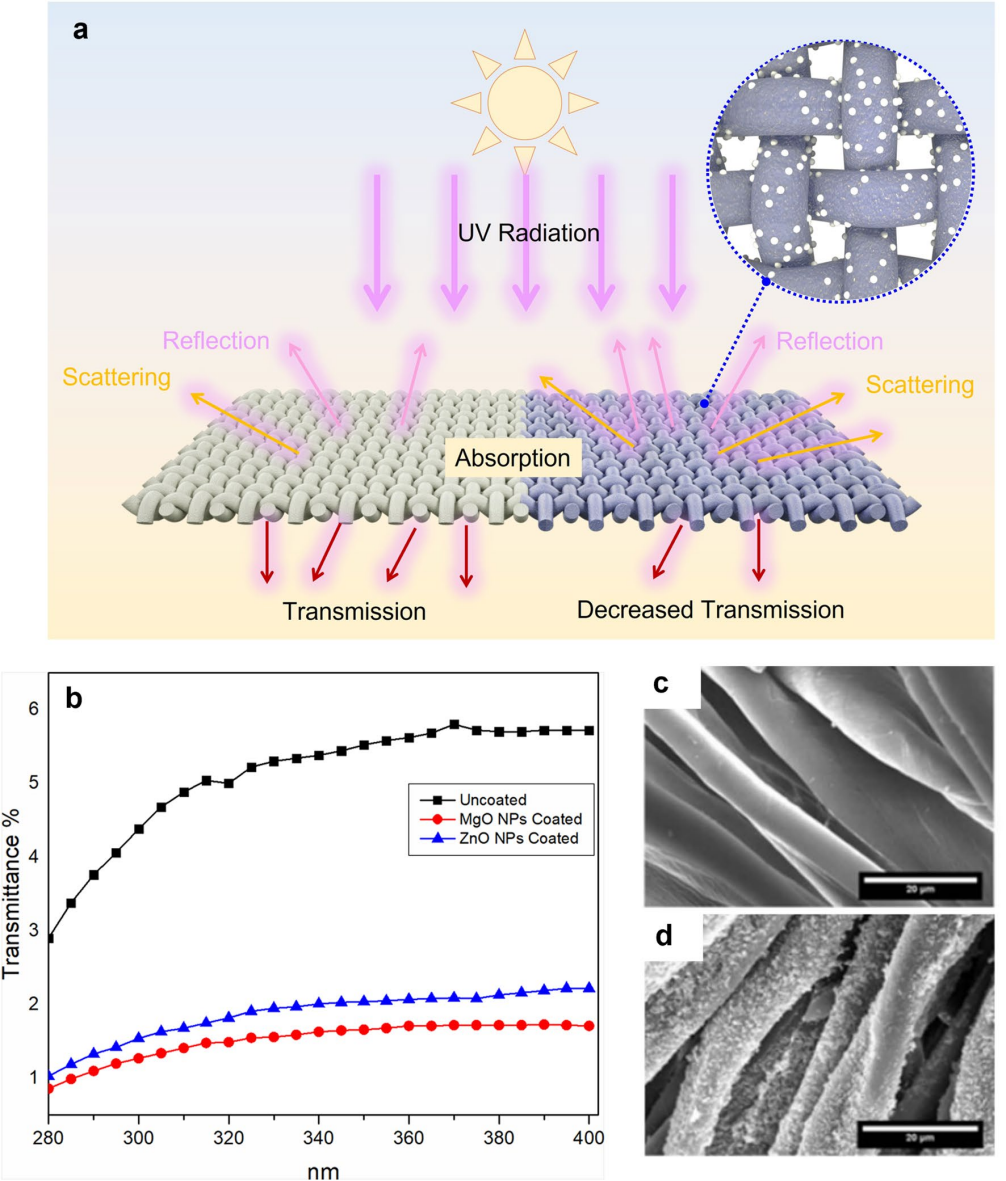
**a** Illustrated ultraviolet (UV) shielding mechanism of the textile fabric treated with oxide nanoparticles (right) by scattering, absorbing, or reflecting the UV radiation when compared to the untreated fabric (left). **b** UV transmittance spectrum of cotton fabric treated with 2% ZnO NPs, with a crystallite size of 20 nm and an aggregated particle size of 80–85 nm; or 2% MgO NPs with a crystallite size of 9 nm and an aggregated particle size of 30–40 nm. Adapted from [201]. **c**, **d** SEM images of cotton fabric without (**c**) and with (**d**) decorated ZnO nanoparticles. The precursor solution (0.1 M zinc salt) was sonicated with fibres for 1.5 h to obtain the particle density shown in **d**. Scale bar is 20 μm. Adapted from [202]

UV-absorbing oxide particles used either as a nanofiller or surface application to decorate the fibres can decrease the UV transmittance of fabrics incorporating them [193–196], as shown in Fig. 6. Their use is an effective and practical strategy in UV-protective fabrics. As the UV-shielding capacity of the oxide particles is determined by their specific surface area, using nanoscale particles obtains very effective UV protection at low loadings. In general, it is the TiO_2_ [197–200] and ZnO [201–203] that are mostly used as nanoparticles applied to increase the UV-protective properties of fabrics and fibres, although MgO [201] and Ag nanoparticles [204] are also used for UV blocking. An exceptional UV-blocking film is achieved by using TiO_2_ nanoparticles [198]. Table 1 summarises the use of oxide nanoparticles in textile fibres/fabrics and their UV-shielding effectiveness. The high UPF values of the fabric at low nanoparticle loadings (Table 1) illustrates the efficacy of this approach.

Personal thermal-management textiles are considered as next-generation textiles [205, 206] and usually consist of multi-layered fabric constructs [207] that can block UV radiation while transmitting visible and reflecting nearinfrared light [208]. One design approach is to incorporate nanofillers in some of the layers. For example, strong UV reflection was provided when TiO_2_ NPs were used to form a composite woven textile based on poly(lactic acid) fibres in a multi-layer metafabric consisting of two layers [209]. A hybrid film made with cellulose nanofiber (CNF)/antimony tin oxide (ATO) also blocks 91.07% UV radiation, reflecting 95.19% near-infrared (NIR), and transmitting 44.89% visible (VIS) light [208]. Such films or fabrics not only can be used in textiles but also can be applied as window thermal barrier films [208].

### Solar UV radiation generates micro- and nanofibres and particles

Textile fibres in fabric exposed to solar UV radiation for extended periods of time can fragment and, because of the uniaxial orientation of polymer molecules, yield microfibres (MF) or nanofibres (NF) as reported for wool and synthetic fibres [210, 211]. The ecotoxicological effects of MFs are still largely unproven [212–214]. However, there is some concern for ecological effects, given that microplastics by weight were estimated to exceed that of zooplankton in surface waters of the North Pacific sub-tropical circulating ocean current [215].

However, not all types of plastics readily photo-fragment into MFs or NFs. For instance, a laboratory exposure of different types of plastic pellets for a duration equivalent to 44 days of natural weathering in simulated seawater showed that the pellets of HDPE and nylon 6 degraded into MFs, but high impact PS and PP pellets under the same conditions did not generate MFs [216]. However, the study employed a photoreactor with UV-C radiation not found in solar radiation, and whether MFs will be derived from virgin pellets undergoing photodegradation in nature is unclear. Macrofibre-yielding MFs under mechanical stress or photodegradation is to be expected. Wool and synthetic textile fibres (~ 2 mm long) exposed to simulated solar (Xenon source) radiation in natural seawater (for a duration equivalent to 1.5 years of natural exposure) showed such fragmentation [211]. Scanning electron micrographs showed evidence of fragmentation into microfibres of short fibres of PET, and those of wool, in less than two months of exposure to the simulated solar radiation. The exposure to the simulated solar radiation also resulted in the release of additive chemicals such as stabilisers and degradation products of PET, including glycol and terephthalic acid [211]. Generally, leached additives from plastics will also be prone to UV degradation, but the rates of their photoreaction and toxicity of any intermediates are unknown. While the UV wavelengths primarily responsible for this fragmentation are unknown, UV-B radiation is a good candidate, since it promotes oxidation of plastics. MFs can also be generated without the involvement of UV radiation, due to mechanical stress, for instance during laundering [217–220]. It is worth noting that the widespread use of face masks and personal protective equipment during the spread of COVID-19 aggravated the plastic fibre pollution and biomedical waste worldwide [221–224].

A similar environmental issue is posed by nanocomposite materials including composite fibres, where the release of nanoscale particles can occur during use (especially working) and with weathering [225]. For example, PP-CNT nanocomposites loaded with MWCNTs released both carbon nanofiller, plastic fragments, and metal (used as a catalyst in nanotube manufacture) when exposed to simulated solar radiation and subjected to mechanical stress [226].

### Role of UV radiation in the fate of micro- and nanoplastics in the environment

It is well established that solar UV radiation facilitates the fragmentation of plastic litter in land and aquatic environments. With micro- and nanoscale plastics reported in food, water, human blood, and placenta in recent studies, this role of UV radiation is receiving close attention. Photodegradative chemical changes on the surface of plastics also influence the diversity and rate of foulant species settling on them in aquatic environments. The composition of the foulant layer determines the rates of biodegradation and especially biomineralisation, and these processes are modified by UV radiation. The sorption of environmental pollutant species including antibiotics, endocrine disruptor chemicals, and metals by plastic debris may also be modified by UV radiation.

## Knowledge gaps

Even though the degradation of plastics on exposure to solar UV radiation has been widely studied, some fundamental questions remain unanswered. Data on how the common additives used in plastic compounds affect the action spectra of deterioration of key properties of materials, and how they change the dose–response of those properties, are not available. Another knowledge gap is the synergistic effect of UV radiation and other weathering agents (e.g. moisture, heat, and air pollutants). Furthermore, reciprocity data that would help better relate the results of accelerated weathering to weathering in outdoor service environments are scarce. In wood materials, the chemistry of UV-induced degradation is relatively better understood but the variability between the diverse commercial wood species has not been adequately explored. An especially promising area of investigation might be the wood-derived antioxidants and UV-stabiliser compounds that can serve as sustainable replacements for the current conventional, often toxic additives.

Use of nanoscale pigments in plastics, textiles, and coatings has been increasingly advocated to protect these materials from UV radiation and to improve other properties. However, disposal and release of nanomaterial during use and at the end of service life remains a concern. The environmental effects of these nano materials are still not clear.

## Conclusions

The service lives of materials routinely used outdoors are limited by the rate of their solar UV radiation-induced degradation as well as by ambient temperatures, and are therefore closely linked to the amounts of exposure to solar UV radiation and climate change. Long service lifetimes of outdoor materials are critical in photovoltaic energy technologies and in building construction. While efficient UV-stabiliser technologies are available to address weathering degradation of materials such as wood and plastics, they invariably add to the lifetime costs of the relevant products. The role played by the Montreal Protocol as well as any measures on climate change mitigation are, therefore, directly pertinent to economic use of materials. Emerging UV-screening technologies for use in plastics and in wood coatings assessed in this assessment offer promise, but often require further techno-economic validation before commercialisation.

Development of high-efficiency UV-protective fibres is also an important aspect of the effect of solar UV radiation on the use of materials. There has been considerable progress on developing fibres with high UPF values for textiles since our last Quadrennial Assessment [227]. Again, their commercialisation requires information on launderability, retention of UPF, and potential release of nanoparticle additives into the environment. With the use of nanocomposites and coatings that incorporate nanoscale pigments, potential release of nanoscale particles during use or disposal of the products is an emerging concern. When plastics undergo UV-facilitated weathering they undergo fragmentation releasing microplastics and nanoplastics into the environment.

## Montreal Protocol and the sustainable development goals

Several of the United Nations Sustainable Development Goals (SDGs, adopted as part of the 2030 Agenda, [228]) are aligned with the Montreal Protocol and its Amendments aiming at protection of the stratospheric ozone layer and mitigation of climate change through phase-out of ozone-depleting substances and their replacements, many of which have large global warming potential. Issues addressed in this assessment are connected to sustainability of energy production (SDG 7: *Affordable and clean energy/Goal 7a*) and safety of the built environment (*SDG 9: Industry, innovation, and infrastructure/Goals 9.1 and 9.4*). In addition, they contribute to evidence-informed policies to be followed when formulating sustainable strategies for production of materials that are needed for our everyday commodities and the infrastructure around us (*SDG 17: Partnership for the goals/Goal 17.14*).

## Data Availability

All data generated or analysed are included.
